# Experimental Study on the Bone Morphogenetic Protein 1-Modified Bone Marrow Mesenchymal Stem Cell Sheets to Promote Mandibular Distraction Osteogenesis

**DOI:** 10.3389/fsurg.2021.786351

**Published:** 2022-02-09

**Authors:** Zhong-Ping Su, Lei Tian, Hong-Tao Shang, Yong Yang, Jin-Biao Lu, Yong-Jie Kang, Li-Sheng He, Jin-Long Zhao

**Affiliations:** State Key Laboratory of Military Stomatology, Department of Oral and Maxillofacial Surgery, National Clinical Research Center for Oral Diseases, Shaanxi Clinical Research Center for Oral Diseases, School of Stomatology, The Fourth Military Medical University, Xi'an, China

**Keywords:** distraction osteogenesis, bone marrow stromal stem cells, bone morphogenetic protein-1, cell sheet, osteogenic efficiency

## Abstract

**Objective:**

The present study aims to increase the concentration of genetically modified bone marrow mesenchymal stem cells (BMSCs) in the distraction osteogenesis (DO) interstitial space and induce the conversion of BMSCs to osteoblasts to improve the osteogenic efficiency in DO and shorten the treatment period.

**Methods:**

Bone morphogenetic protein 1 (BMP-1) and green fluorescent protein (GFP) gene-modified cell sheets of BMSCs were constructed by tissue engineering. Thirty-six New Zealand white rabbits were randomly divided into three groups: group A (the blank control group), group B (the GFP group) with the injection of GFP gene-modified BMSC sheets into the DO gap, and group C (the BMP-1 group) with the injection of BMP-1 gene-modified BMSC sheets into the DO gap. Rabbits in all three groups were distracted for 5 days at a distraction rate of 2.0 mm/d, once/day. After distraction, the above-mentioned cell sheet suspension was injected into the distraction gap to observe osteogenesis, which was observed by gross specimen observation, micro-computed tomography (Micro-CT) scanning, and histomorphology.

**Results:**

The gross specimen observation showed that all animals had smooth and continuous bone cortex in the distraction region with relatively high hardness. The osteogenesis quality or hardness was ranked from the highest to the lowest, as Group C > Group B > Group A. Micro-CT and histomorphological observation revealed that group C had better maturation and bone volume of the new bone in the DO region at weeks 3 and 6 than groups B and A.

**Conclusion:**

BMP-1 gene-modified BMSC sheets could effectively promote the formation of new bone during rapid DO in the mandible, compensating for the poor osteogenesis caused by rapid distraction and providing a new approach to shorten the DO treatment period in clinical practice.

## Introduction

Oral and maxillofacial defects and deformities caused by tumors, trauma, infection, congenital malformations, etc., seriously affect the psychological health and quality of life in patients. Due to the unique requirements of maxillofacial anatomy and oral function, the repair and reconstruction of maxillofacial deformities and defects have always been the hot spots and complex areas of concern in oral and maxillofacial surgery. For jaw defect deformities, the commonly used repair methods in clinical practice include autologous bone grafting, the distraction osteogenesis (DO) technique, and individualized repair prosthesis, among which autologous bone grafting occupies a dominant position in bone defect repair. However, the complexity of the autologous bone grafting procedures and the high technical requirements have limited the clinical application (1). Compared with other jaw defect repair methods, the DO technique has many advantages, such as relatively simple surgery, no need to open a second surgical area, and soft and hard tissues can be extended simultaneously, so it is widely adopted in the repair and reconstruction of various craniomaxillofacial defects (2, 3). However, due to the different skeletal structures in various parts of the maxillofacial region, the poor quality of osteogenesis at rapid distraction rate, and the long period of distraction severely limit the clinical application. Therefore, how to improve the quality of osteogenesis and shorten the distraction period is the key problem faced in the clinical application of DO.

Numerous studies have shown that mesenchymal stem cells (MSCs) have multidirectional differentiation potential, among which they can differentiate into osteoblasts and promote new bone formation (4–6). Therefore, can MSCs have a role in promoting new bone formation in the process of DO? Ma et al. (7) injected the bone marrow mesenchymal stem cell (BMSC) sheets into the DO gap of the mandible in rabbits with the adoption of a tissue engineering method. The results showed that BMSCs had a good effect on promoting new bone formation, but the osteogenic efficacy should be further improved. Therefore, further studies are needed to investigate how to further stimulate the osteogenic potential of BMSCs, improve the osteogenic quality of DO, and shorten the distraction period.

Bone morphogenetic proteins (BMPs), an important class of osteogenic growth factors, have been shown to play an essential role in bone reconstruction and regeneration (8). In a bone defect repair experiment, BMSC sheets transfected with BMPs were transplanted into the bone defect area and effectively promoted new bone formation (9). Currently, among the more than 20 BMPs identified, BMP-2, BMP-4, and BMP-7 have been shown to play an essential role in the osteogenesis process (10–12). While BMP-1, an important bone morphogenetic factor in the BMPs family, is the basis for extracellular matrix development and formation and affects bone scab formation and bone regeneration by regulating collagen deposition (13, 14). At present, little research has been reported on BMP-1, and only a few studies have found that it has a bone regeneration-promoting effect in fracture healing (15). Whether BMP-1 can be transfected into BMSCs to promote new bone formation during DO remains to be confirmed by the investigation.

Based on the above hypothesis, the present study attempted to construct the BMP-1-transfected cell sheets of BMSCs through a combination of tissue engineering and gene transfection techniques and then transplanted them into the distraction gap of the mandible in rabbits to explore their role in promoting new bone remodeling and mineralization in DO.

## Materials

Experimental materials used for cell culture were referred to as those in experiment 1. Inveon Micro CT machine (Siemens, Germany), Inveon Acquisition Workplace three-dimensional (3-D) reconstruction processing software, and Inveon Research Workplace dedicated skeletal analysis software. Upright microscope (Leica DM6000B, Germany), general surgical instruments (Shanghai Medical Equipment Co. Ltd., China). Bone power system (bone saw, bone drill, wires, and pedals from Crown Eagle, Guangzhou, China). Built-in osteogenesis distractor (Zhongbang Co., Xi'an, China).

## Methods

### Cell Culture and Construction of Cell Sheets Transfected With BMP-1

Cell culture and construction of cell sheets transfected with BMP-1 were conducted as described previously (16).

### Construction of a Rabbit Mandibular DO Model

**Figure 1:**
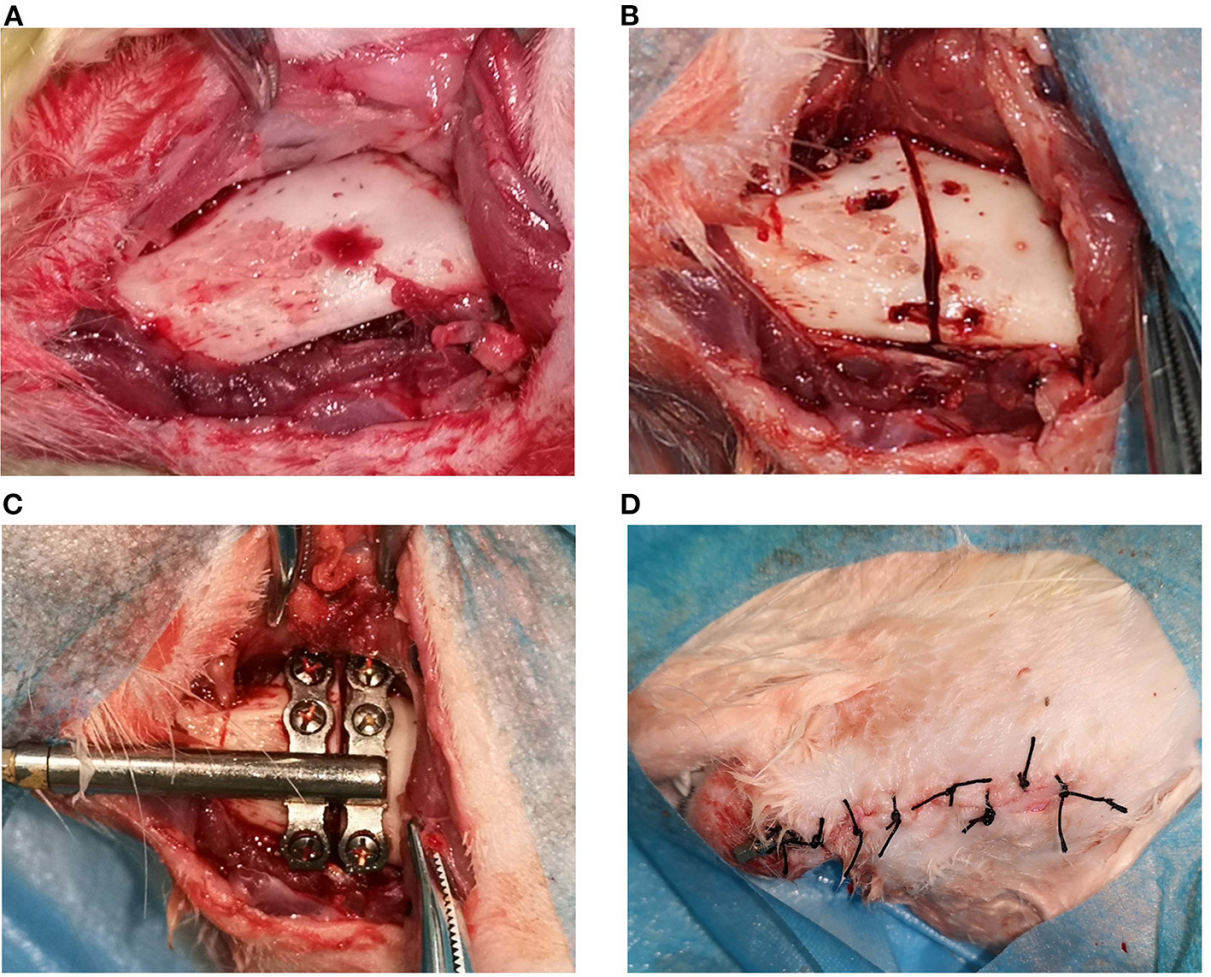
The operation procedures of distraction osteogenesis: **(A)** demonstrates the exposed bone surface during incision; **(B)** shows the truncation of the bone cortex; **(C)** illustrates the installation of the distractor; Figures 1–4 shows the incision suture.

In this experiment, a 3-month-old New Zealand white rabbit was used as our experimental animal. All experimental rabbits were subjected to left mandibular osteotomy in the experiment. The experimental animals were given 0.1 g/kg of cefazolin intramuscularly 30 min before surgery to prevent postoperative infection. Xylazine mixed with sodium pentobarbital was injected intramuscularly. After anesthetizing, the right face of the rabbit was placed close to the workbench, and the left face was fixed in a lateral position facing upwards on the operating table. The skin was prepared on the left submandibular area, wiped clean with gauze, disinfected with 3% iodine, and covered with disposable sterile cloth sheets. The surgical incision was located 1.5 cm from the lower edge of the left mandible. After the onset of local infiltration anesthesia with 5 ml of 2% lidocaine hydrochloride, the skin, subcutaneous tissues, and platysma were incised in sequence along the designated incision line ~4 cm in length. The submandibular gland was ligated subcutaneously (to prevent postoperative wound salivation). The flap was then turned upward along the inferior edge of the platysma to expose the lower edge of the mandible, and the periosteum was incised along the inferior edge of the mandible. The flap was turned upward to expose the buccal side of the left mandible with the adoption of a periosteum dissector and with the protection of the mandibular nerve during the process, strip the periosteum as little as possible without affecting the exposed area (as shown in Figure 1A).

The built-in titanium distractor used in the present experiment was supplied by Zhongbang Co. (Xi'an, China). In the current experiment, the osteotomy line was designated between the first and second mandibular molars, the anterior end of the distractor was oriented in the proximal-middle direction, and the distractor fixation wings were properly shaped so that the bottom surface of the distractor would fit the lateral surface of the mandible. A micro-drill was used to drill the hole, the distractor was fixed with a micro-6 mm titanium nail so that the gap between the anterior and posterior fixation wings was precisely between the first and second mandibular molars, and a mark was made between the fixation wings using the matching reciprocating saw. The titanium nail on the fixing wing was removed, then the reciprocating saw was used to cut the inferior edge of the mandible and the buccal bone cortex. The bone chisel was adopted to bluntly separate the lingual bone cortex. The bone cortex was cooled down by normal saline rinsing during osteotomy to avoid high local temperature (as shown in Figure 1B). Finally, the distractor was re-fixed with micro screws in the position designed before the osteotomy. With the completion of the fixation of the distractor, the extension lever could open the osteotomy gap without resistance, indicating that the distractor was successfully built-in (as shown in Figure 1C). After flushing the wound with plenty of normal saline and adequate hemostasis, the wound was closed in layers (as shown in Figure 1D). The front end of the distractor connected to the extension lever was led out from the incision to facilitate the application of lateral force. Erythromycin ointment was applied to the wound. Finally, the crowns of the upper and lower incisors were ground short.

With the full recovery of consciousness, the experimental animals were sent back to the animal room. Then, the animals' mental state, dietary situation, and weight were observed and recorded daily. Postoperatively, cefazolin was injected intramuscularly to prevent infection, and the wound was disinfected with iodophor daily for 1 week. Liquid food was supplied on the first day after surgery, soft food on the second to the sixth day, and gradually normal feed was given after 1 week, according to the recovery condition.

### Experimental Grouping and Distraction Intervention

**Figure 2:**
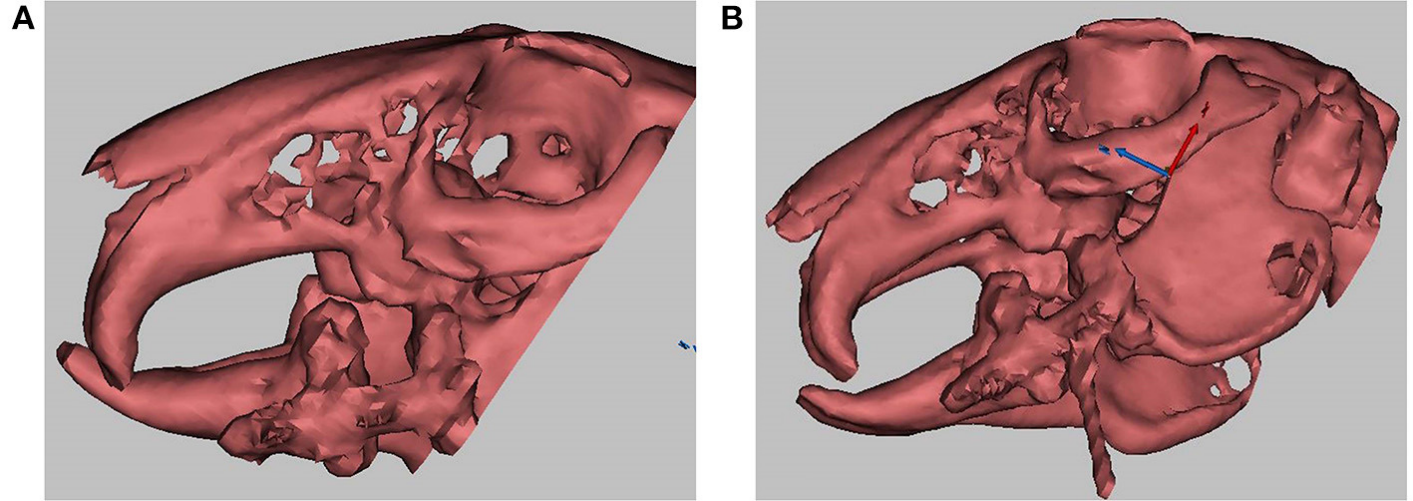
**(A)** demonstrates that the distraction length achieved the experimental expectation (1 cm), and the arrow indicates the distraction gap; **(B)** shows a real-time computed tomography (CT)-guided distraction injection, and the arrow indicates the syringe needle.

Thirty-six New Zealand white rabbits were randomly divided into three groups, with 12 rabbits in each. Group A (the blank control group): the distraction rate was 2.0 mm/d for 5 days, and normal saline was injected into the distraction gap. Group B (the green fluorescent protein [GFP] group): the distraction rate was 2.0 mm/d for 5 days, and the GFP gene-modified BMSC sheets were injected into the distraction gap. Group C (the BMP-1 group): the distraction rate was 2.0 mm/d for 5 days, and the BMP-1 gene-modified BMSC sheets were injected into the distraction gap. After a 3-day delay, groups A, B, and C were distracted for 5 days at a rate of 2.0 mm/d, once/d, and the total distraction length was 10 mm. A 3-D CT scan was performed at the end of the distraction, and the distraction length was observed to be as expected (as demonstrated in Figure 2A, the distraction gap indicated by the arrow was ~1 mm in length).

**Figure 3:**
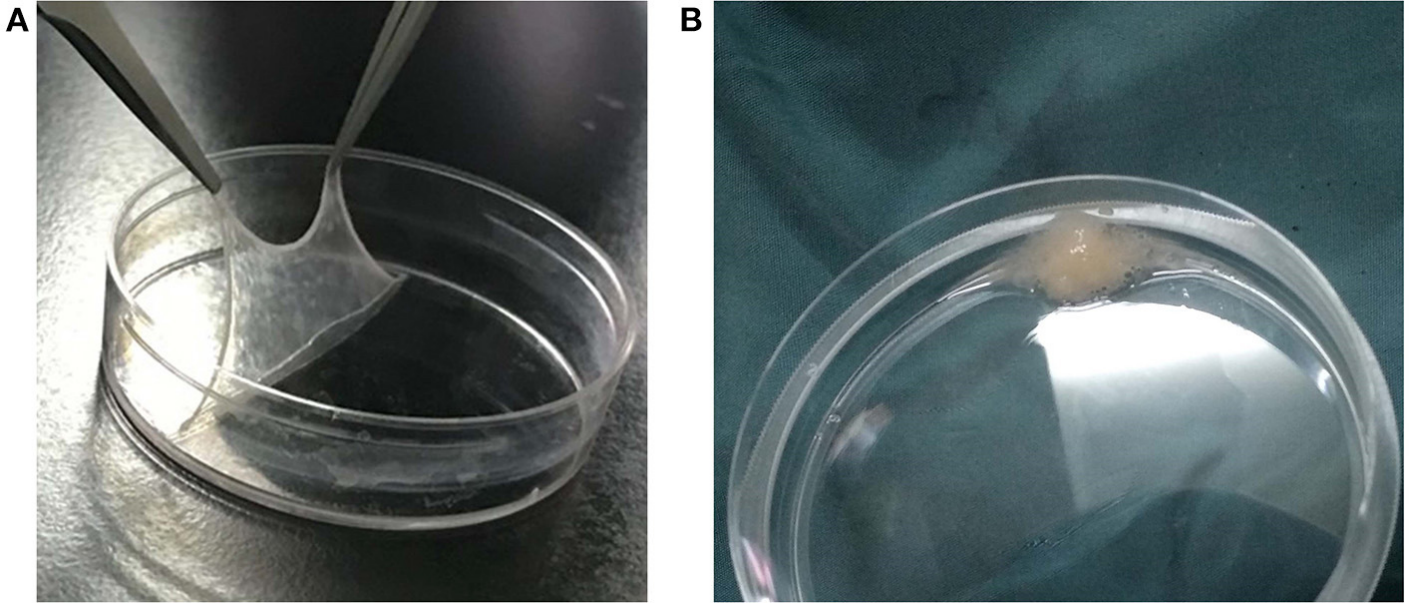
Bone marrow mesenchymal stem cell (BMSC) sheets and sheet fragment suspension: **(A)** is the BMSC sheet; **(B)** demonstrates the BMSC sheet fragment suspension.

The cell sheets from the BMP-1 and GFP groups obtained in experiment 2 (Figure 3A) were made into a cell sheet suspension (Figure 3B). Then, 0.3 ml of normal saline, 0.3 ml of cell sheet suspension from the GFP group, and 0.3 ml of cell sheet suspension from the BMP-1 group were injected into the distraction gaps on the last day of the distraction period in groups A, B, and C, respectively, with the use of a 1 ml syringe with an 18 G injection needle (17, 18). The injection procedure was conducted under CT guidance to ensure injection into the distraction gap (as illustrated in Figure 2B, the arrow indicating the syringe needle). After 3 and 6 weeks of fixation (19), six rabbits in each group were executed for observation, respectively.

### Gross Specimen Observation

After 3 and 6 weeks of fixation, the experimental animals were executed by intravenous injection of an overdose of anesthetics through the ear veins. The animals were then dissected, and the mandibles were completely separated. The amount of bone and mineralization of the new bone in the DO region of the mandible was observed in the gross specimens and then photographed and kept for documentation. In this study, we evaluated the hardness of new bone by observing the texture of new bone and using fine needle puncture.

### Micro-CT Scanning Analysis

After 3 and 6 weeks of fixation, the rabbits were executed by an intravenous injection of an anesthetic overdose through the ear veins. The dissected mandibular specimens were fixed in 4% paraformaldehyde for 24–48 h, washed under running water, and scanned by micro-computed tomography (Micro-CT) (with a layer thickness of 10 μm). Finally, the scanned data were reconstructed by the Inveon Acquisition Workplace 3-D reconstruction processing software and analyzed by the Inveon Research Workplace special skeletal analysis software. The obtained results of BV/TV (new bone volume/total new tissue volume), BS/BV (new bone surface area/new bone volume), TbTh (the mean trabecular thickness), TbN (the mean trabecular number), and TbSp (the mean trabecular spacing) were analyzed statistically to determine whether there existed differences among the groups.

### Histological Analysis

With the completion of Micro-CT detection, the experimental specimens were soaked in 4% formaldehyde solution for 24 h and then decalcified with 10% decalcification solution (syringe needle could be pierced smoothly), routinely dehydrated, and sections were paraffin-embedded. The sections were then stained with hematoxylin-eosin (H&E) and Masson's trichrome (Masson's).

### Statistical Methods

All data in the present study were recorded by mean ± standard deviation. The SPSS 19.0 software was adopted for data analysis. One-way ANOVA was adopted to test whether the groups' experimental data were statistically different (*P* < 0.05 is a statistically significant difference).

## Results

### Detection of the BMP-1 Transfection Efficiency Was the Same as Described Previously

#### Gross Specimen Observation

All animals in the present study were able to withstand the relevant experimental operations, and there were no deaths due to the testing. There was no distractor loosening or detachment, no infection in the operation area, and all experimental animals completed the experiment successfully (16).

**Figure 4:**
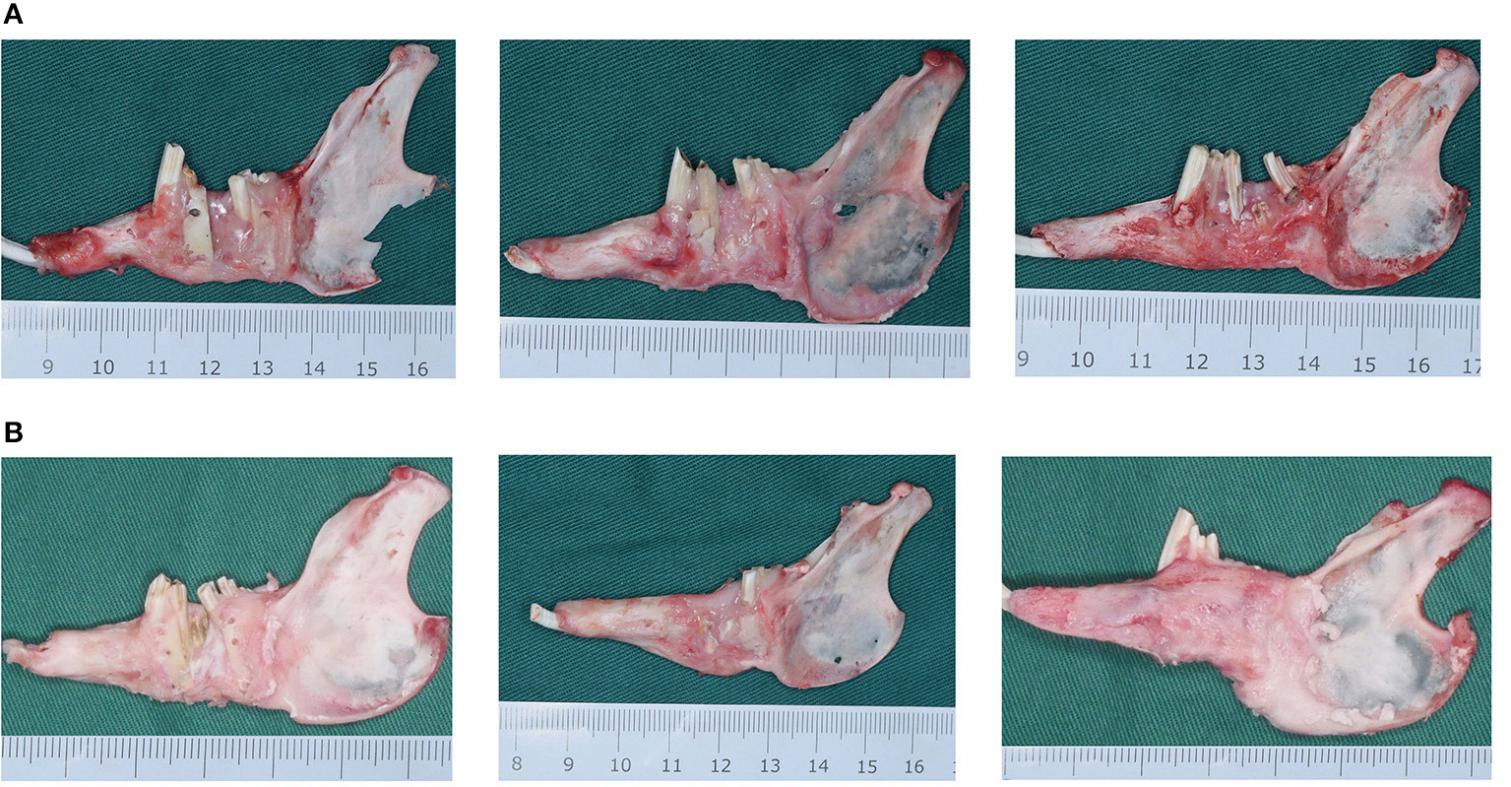
**(A)** The mandibular gross specimens from the blank control (group A), the green fluorescent protein (GFP) (group B), and the bone morphogenetic protein 1 (BMP-1) (group C) at 3 weeks of fixation. **(B)** The mandibular gross specimens from the blank control (group A), the green fluorescent protein (GFP) (group B), and the bone morphogenetic protein 1 (BMP-1) (group C) at 6 weeks of fixation.

In all experimental animals, after 3 and 6 weeks of fixation, the mandible was successfully lengthened, the lower anterior teeth were shifted to the right, and a significant rightward deformity of the lower jaw was observed. At 3 weeks of fixation, it was found that the lingual side of the DO region was significantly better than the buccal side in terms of bone quantity and quality, and the cortex was relatively continuous without obvious depression and had strong hardness in all animals. In group A, the new bone in the DO region was mainly the fibrous connective tissue, and the new bone tissue on the buccal side was concaved inward compared with the normal bone surface in the proximal and distal middle part, and the original osteotomy line could be clearly observed with the continuity of the bone being interrupted. Although the new bone on the lingual side had good continuity, it manifested poor hardness and was reddish, indicating poor calcification. In group B, the new bone in the DO region had begun to ossify, but the degree of ossification was still poor with moderate bone hardness. The buccal side of the new bone surface was slightly lower than the normal bone surface in the proximal and distal middle part. However, the original osteotomy line could still be identified. The hardness and mineralization of the new bone in the DO region in group C were significantly higher than in groups A and B. The bone cortex was smooth and continuous, and the original truncated bone was faintly visible, but the new bone was relatively red in color (as demonstrated in Figure 4A). After 6 weeks of fixation, the hardness of the new bone in the mandibular distraction region was significantly higher than at 3 weeks. In group A, depression of the buccal cortex was still visible, the scope of the depression was smaller, and the texture of the bone at the depression was harder than at 3 weeks, with discontinuous bone cortex and the proximal and distal middle osteotomy lines were clearly visible. In group B, no significant depression was visible in the buccal bone cortex with continuous cortex, but the truncation line could still be identified near the top of the alveolar ridge. In group C, the buccal bone cortex in the distraction region was continuous and smooth, the truncation line could not be distinguished, and the hardness and color of the bone were basically the same as those of the proximal and distal host bones (Figure 4B).

### Results of Micro-CT and Data Analysis

**Figure 5:**
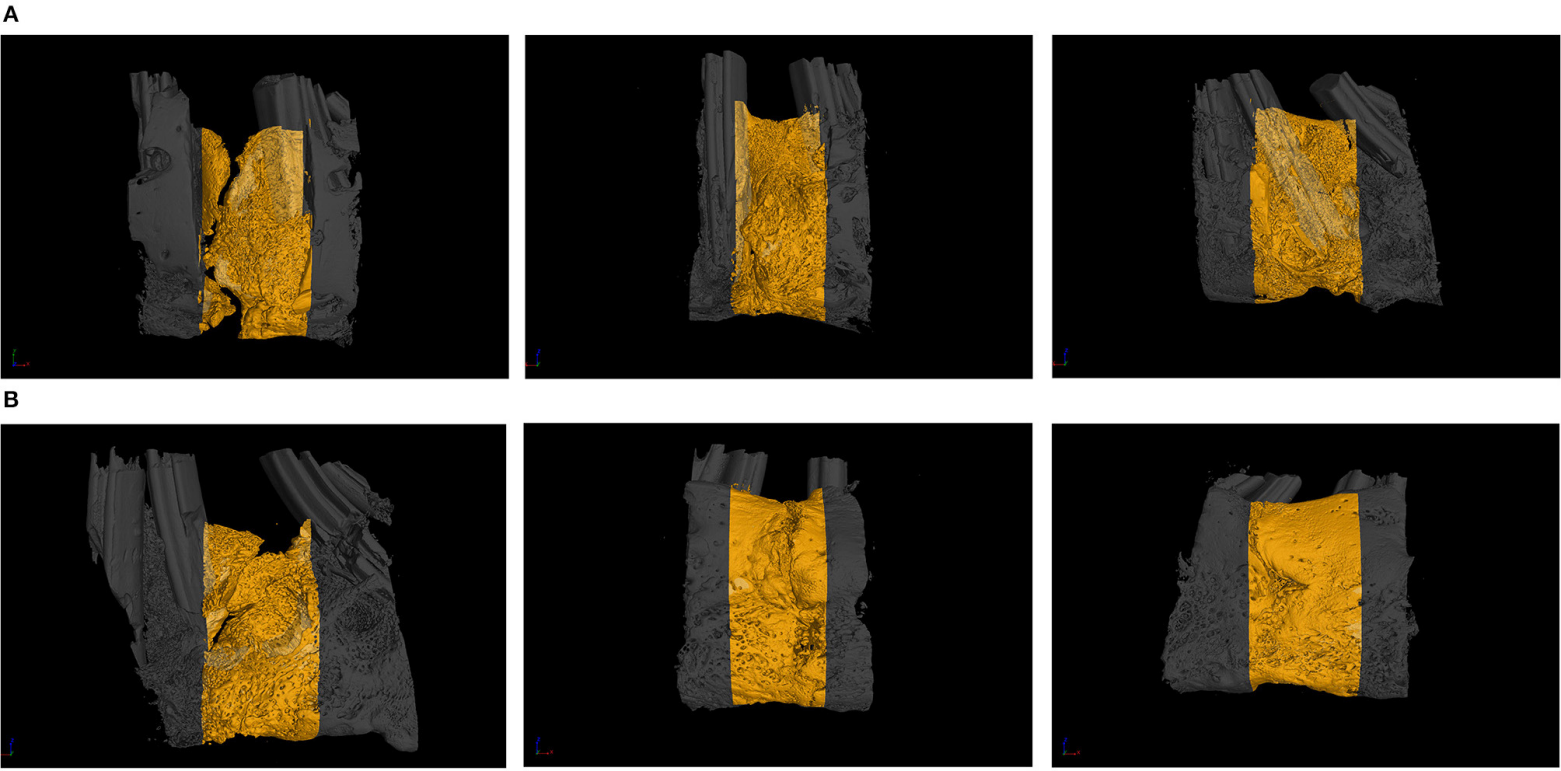
**(A)** The Micro-CT (micro-computed tomography) from the blank control (group A), the green fluorescent protein (GFP) (group B), and the bone morphogenetic protein 1 (BMP-1) (group C) at 3 weeks of fixation (the yellow indicates the ontogenetic region). **(B)** The Micro-CT (micro-computed tomography) from the blank control (group A), the green fluorescent protein (GFP) (group B), and the bone morphogenetic protein 1 (BMP-1) (group C) at 6 weeks of fixation (the yellow indicates the ontogenetic region).

The images obtained from the Micro-CT scanning of the mandibles at weeks 3 and 6 of the experiment are shown in Figure 5, and the results obtained were generally consistent with the gross specimen observations. The proportion of osteogenesis in the lingual cortex of the mandible was significantly higher than the buccal side in all groups. At week three, group A had poor osteogenesis with less bone formation, discontinuity of the newly formed bone, and obvious bone gaps were visible. Group B had no fissures in the new bone, but the bone on the buccal side was depressed, and the bone density was poor. Compared with those in groups A and B, the cortex on the buccolingual side in group C was more mature in morphology and texture, with a higher amount of osteogenesis and ossification at week 3 (as shown in Figure 5A). At week 6, all three groups showed significant improvement in the quality of new bone. In group A, bone quality and quantity were improved, but bone fissures were still visible near the alveolar ridge, and the bone was still osteoporotic. In group B, the bone volume and texture of the new bone were close to normal, but traces of the osteotomy line were faintly visible near the alveolar ridge, and the bone cortex was slightly rough. In group C, the bone cortex was smooth and continuous. The truncation line was completely indistinguishable, and the osteogenic volume and bone density were significantly better than in groups A and B (as demonstrated in Figure 5B).

**Figure 6:**
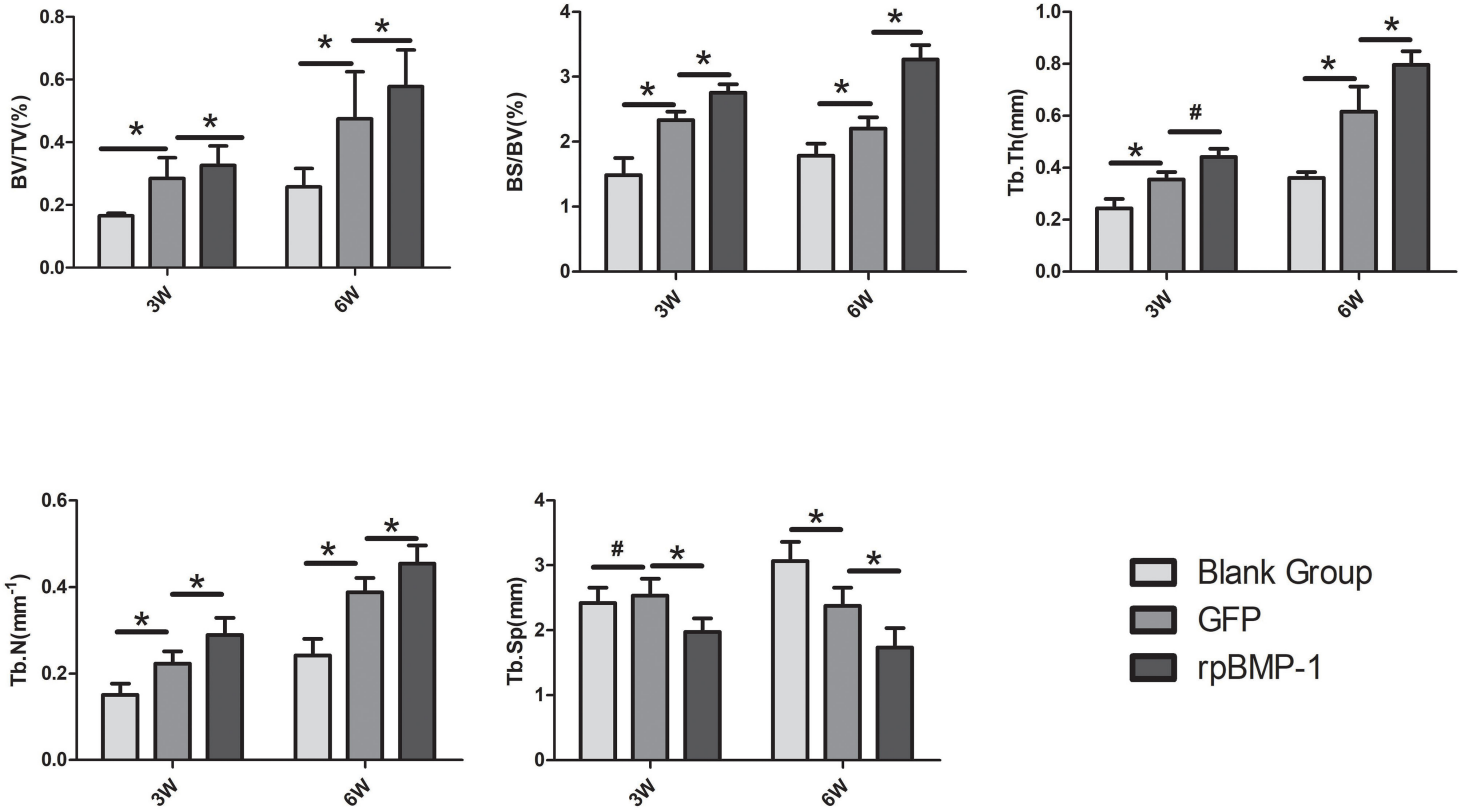
Quantification analysis of osteogenic indicators in the distraction osteogenesis (DO) region of the mandible by Micro-CT (micro-computed tomography) in the blank control (group A), the green fluorescent protein (GFP) (group B), and the bone morphogenetic protein 1 (BMP-1) (group C) at three and six weeks of fixation. TbTh, the mean trabecular thickness; TbSp, the mean trabecular spacing; BS/BV, the new bone surface area/new bone volume; BV/TV, new bone volume/total new tissue volume; TbN, the mean trabecular number. *P < 0.05; ^#^*P* > 0.05.

The following results were obtained from the quantitative analysis of the Micro-CT indicators (as shown in Figure 6). At weeks 3 and 6, BV/TV, BS/BV, TbTh, and TbN in group C were better than in groups A and B, and the above indicators in group B were better than in group A. Comparisons by ANOVA and least significant difference (LSD) t-test (A–B, B–C) showed that the above differences (group A vs. group B, group B vs. group C) were statistically significant (*P* < 0.05). TbSp increased slightly in group B compared with group A at week three, but the difference was not substantial, and there was no statistically significant difference (P > 0.05). TbSp decreased more in group C than in group B. TbSp at week 6 were lower in group C than in both groups A and B, and lower in group B than in group A. There existed statistically significant differences by the comparisons through ANOVA and LSD t-test (group A vs. group B, group B vs. group C) (*P* < 0.05). This was because more mature new bone and more trabeculae would result in an increased bone density and a smaller distance between trabeculae.

### Histomorphological Observation and Quantitative Analysis

**Figure 7:**
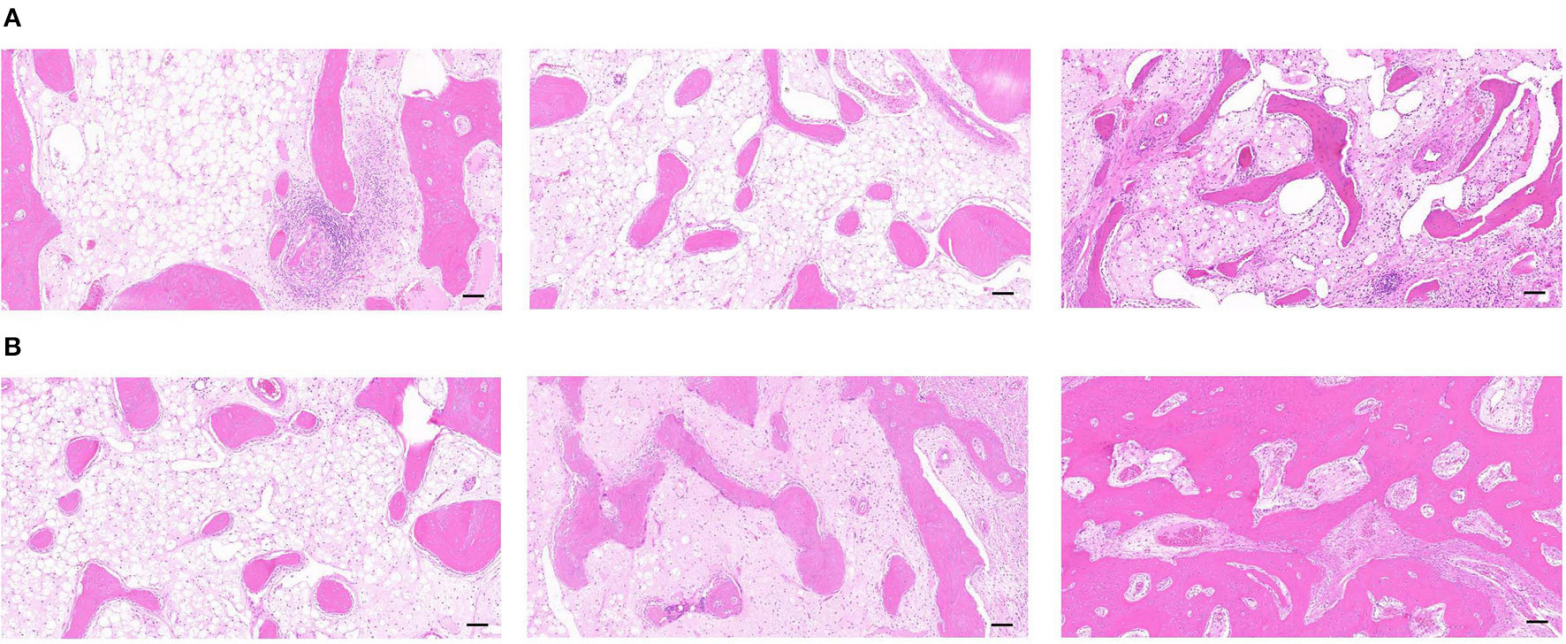
**(A)** The H&E staining of the distraction osteogenesis (DO) region of the mandible in the blank control (group A), the green fluorescent protein (GFP) (group B), and the bone morphogenetic protein 1 (BMP-1) (group C) at 3 weeks of fixation (Scale: 100 μm). **(B)** The H&E staining of the distraction osteogenesis (DO) region of the mandible in the blank control (group A), the green fluorescent protein (GFP) (group B), and the bone morphogenetic protein 1 (BMP-1) (group C) at six weeks of fixation (Scale: 100 μm).

As observed by the H&E staining of the new bone in the DO gap, groups A and B had similar maturation of new bone and less vascular components after 3 weeks of fixation. However, the content of the new trabeculae was significantly higher in group B than in group A. Meanwhile, apparent trabeculae formation was observed in group C with well-developed trabecular structures, and a higher number of red blood cells could be observed in the bone marrow cavity, which indicated a good blood supply (Figure 7B). There was a significant increase in the number of trabeculae in the new bone at week 6 compared with week 3, as well as an increase in the maturity of the trabeculae, which became thicker and less distant from each other, which were consistent with the results observed by Micro-CT. In addition, the content and maturity of trabeculae in group C were significantly better than in groups A and B. A more mature bone marrow cavity was visible in the center of the mature trabeculae, and more new blood vessel components were visible inside (Figure 7B).

**Figure 8:**
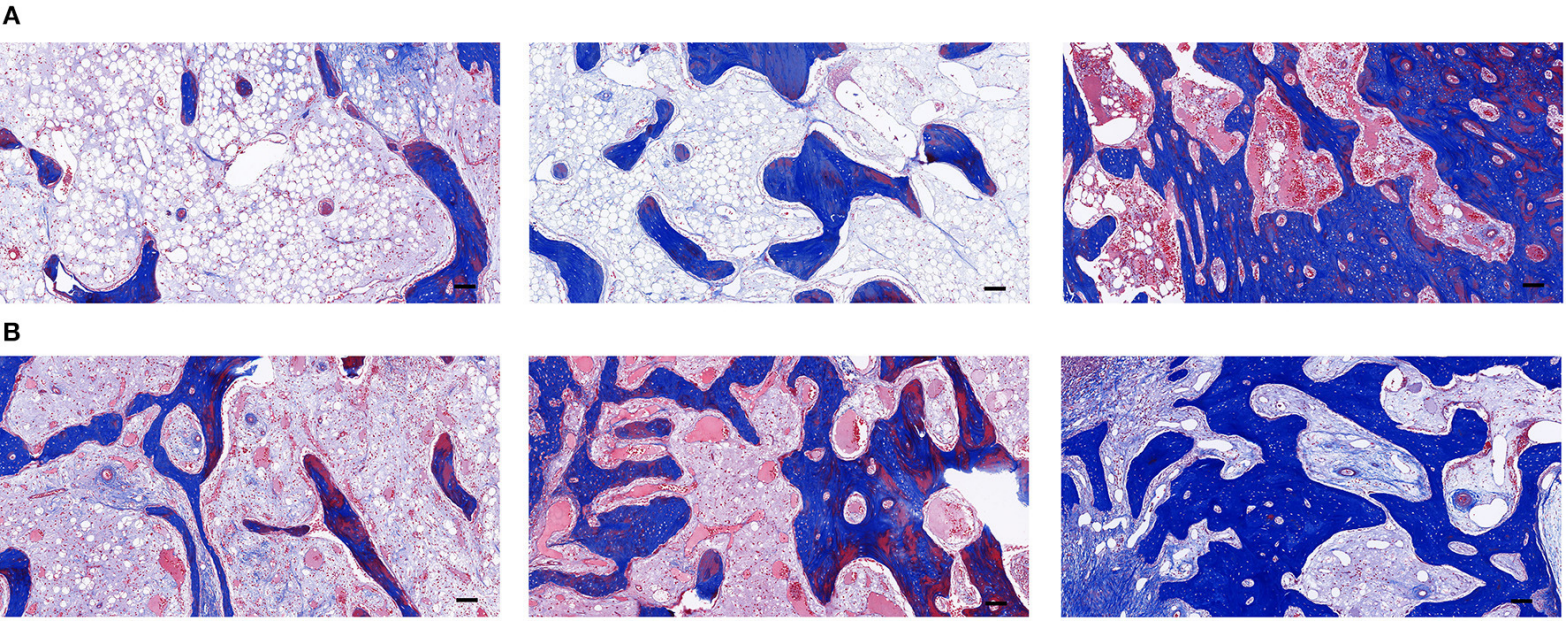
**(A)** The Masson's staining of the distraction osteogenesis (DO) region of the mandible in the blank control (group A), the green fluorescent protein (GFP) (group B), and the bone morphogenetic protein 1 (BMP-1) (group C) at three weeks of fixation (Scale: 100 μm). **(B)** The Masson's staining of the distraction osteogenesis (DO) region of the mandible in the blank control (group A), the green fluorescent protein (GFP) (group B), and the bone morphogenetic protein 1 (BMP-1) (group C) at six weeks of fixation (Scale: 100 μm).

Masson's staining of the new bone showed that a few blue-stained collagen fibers were visible in groups A and B at week 3 of fixation, and the maturity of new bone was similar in both groups but with poor osteogenesis, and a few vascular components were visible inside, which were consistent with the results by H&E staining. More blue-stained collagen fibers were visible in group C. The collagen fibers were better than those in groups A and B in terms of number and maturity (Figure 8A). At week 6 of fixation, the amount and maturity of collagen fibers in groups A, B, and C were elevated compared with those at week 3 with the emergence of red-stained collagen fibers. In group C, the nearly matured bone marrow cavity was surrounded by collagen fibers with gray-white calcified nodules being visible among the fibers, suggesting that the new bone in group C was significantly better than those in the other two groups in terms of osteogenic volume and degree of mineralization (Figure 8B).

## Discussion

DO is a surgical technique to treat skeletal deformities or defects by lengthening or expanding the bone, which is achieved by applying a certain amount of tension to the bone segment where the periosteum and soft tissues are attached. DO has become an effective tool for treating various craniomaxillofacial deformities and bone defects, such as short craniofacial deformities, temporomandibular joint ankylosis, bone defects after tumor resection, etc. (20, 21). However, the main drawback of this method is the long treatment period and the poor quality of bone formation at rapid distraction rate, which seriously limits further clinical applications. Therefore, the current investigation is focused on how to rapidly improve the quality of osteogenesis and shorten the treatment period.

A retraction rate of 1 mm/d is adopted at present in most clinical practices to achieve distraction to ensure good osteogenesis. However, it has been reported in the literature that prolonged slow distraction will induce pain and increase the risk of local infection (22, 23). Although increasing the distraction rate may be an effective way to shorten the distraction period, quick distraction may result in poor osteogenesis of new bone. Therefore, how to solve the adverse effects of rapid distraction has become a hot topic of research for surgeons in the field of craniomaxillofacial surgery.

During the process of DO, recruitment, colonization, and clonal expansion of primitive MSCs under appropriate mechanical stimulation, followed by differentiation toward osteoblasts, are vital steps in the regeneration of new bone in the distraction region (24, 25). Poor bone regeneration in the distraction region may be due to insufficient migration, proliferation, or differentiation of osteogenic progenitor cells in the distraction region. This condition is particularly prone to occur in patients whose original MSCs have been reduced or after severe trauma or radiotherapy (26) and in patients whose host tissue beds have been severely damaged. Therefore, supplementation of osteoblasts with autologous BMSCs is a more reasonable way to promote osteogenesis to ensure ideal bone regeneration in the distraction region when the systemic conditions are poor or when more osteogenesis is needed. In recent years, some researchers injected the BMSC suspension into the DO interstitial space to accelerate the formation of new bone, but the BMSC suspension is susceptible to re-absorbing and diffusing, and it is difficult to get it to stay in the osteogenic site; thus, the therapeutic efficacy is minimal. With the development of regenerative medicine, gene transfection technology and tissue engineering have shown promising applications in shortening the DO treatment period. Therefore, in the present study, the BMP-1-modified cell sheets of autologous BMSCs were constructed by combining the tissue engineering and gene transfection techniques and then were transplanted into the distraction gap of the mandibles in rabbits to explore the role in new bone formation and shortening the treatment period in DO.

In the present study, the BMP-1 gene therapy was adopted, and it confirmed the osteoinductive effect of BMP-1 on mandibular DO. By gross specimen observation, Micro-CT, and histomorphological observation, it was found that new bone could be formed faster with BMP-1-modified BMSC sheets compared with those with the adoption of the GFP, suggesting that gene therapy with BMP-1 *in vitro* could promote new bone formation in DO. Since efficient transfection of BMSCs with BMP-1 is key to the process, we examined the transfection efficiency by fluorescence microscopy and immunoblotting (western blot, WB) as described previously (16), and over-expressed BMP-1 protein was detected in the culture supernatant of BMSCs transfected with BMP-1, which indicated a high transfection efficiency of BMSCs.

To make the DO model more clinically relevant, the mandibular DO osteotomy line was designated between the mandibular first and second molars. The height and thickness of the mandible in the posterior teeth region in the rabbit are increased compared with those in the anterior teeth region, and there exists interference from the teeth. Thus, the difficulty of the operation and the requirements of DO are increased a great deal, but the present experimental design was undoubtedly closer to the clinical reality and would have more significance for clinical guidance.

In order to reduce the infection rate of experimental animals, personalized built-in tractors were also used in this experiment. Previously, most of the distractors used in DO were external, which were easy to apply force, but they were easy to loosen or detach with a relatively high risk of infection. In the DO model of the rabbit mandible constructed by Ma et al. (27), although the built-in distractor was adopted, the exposed part of the extension lever was placed inside the mouth. This design interfered with the feeding of the animal on the one hand, and it was difficult to apply force each time, even it should be conducted under anesthesia. On the other hand, saliva could easily enter the distraction region through the intraoral wound and result in infection, which might affect osteogenesis in severe cases. In the present experiment, an individualized built-in distractor was adopted, which was fixed to the lateral aspect of the mandibular body through the inferior mandibular incision, and the part of the distractor attached with the extension lever was led out through this incision, which was designed to facilitate the forcing of the distractor and also greatly reduced the incidence of postoperative infection. All experimental animals in the present study were able to withstand the experimental operations, including anesthesia, osteotomy, and postoperative distraction, and there were no animal deaths during the experimental procedures. There was no distractor loosening or detachment, no infection in the operation area, and all experimental animals completed the experiment successfully.

Due to the stimulation of slow distraction force, a large number of osteoblasts and endogenous growth factors existed in the distraction gap at the early stage, and exogenous BMP-1 interacted with osteoblasts and endogenous growth factors at the site of bone formation, which can effectively promote osteogenesis. The osteogenesis in DO is closely correlated with the timing of the fixation period, and the most active osteogenesis period is the early stage of the fixation (28). Therefore, selecting the time point to promote new bone formation using genetic techniques is particularly important. Some researchers have achieved satisfactory results in stimulating new bone formation by local injection of BMP-2 at the early stage of DO fixation (29). Therefore, in the present experiment, injection of the BMP-1 gene was conducted at an early stage after the completion of bone distraction, and the results showed that it could effectively improve the quality of new bone in the distraction region, which proves the effectiveness of gene therapy at an early stage with completion of distraction.

In the mandibular DO in rabbits, a latency of 5–7 days, a distraction rate of 0.9–1.0 mm/day and a consolidation period of 2–8 weeks are recommended as the optimal distraction protocol (19, 30), with a higher distraction rate leading to delayed fibrous healing and osteogenesis of the distraction gap. A short latency (three days), a rapid distraction rate (2.0 mm/day, once/day) and a short consolidation period (3, 6 weeks) were adopted in the present study to construct a rapid distraction model and verify the effect of BMP-1-modified BMSC sheets in promoting distraction osteogenesis. The results of the present study showed that with a distraction rate of 2.0 mm/d, group A had poor osteogenesis and bone discontinuity in the distraction gap after 3 and 6 weeks of fixation. The experimental results suggested that a distraction rate of 2.0 mm/d was too fast for mandibular DO. In group B, after the 3- and 6-week fixation, the quality of osteogenesis was slightly better than in group A. However, the amount of osteogenesis on the buccal side was still insufficient. The bone surface was obviously concave compared with the surrounding bone surface, it was rough, and faint osteotomy lines were still visible. Both Micro-CT and histomorphological observations showed that group B had better osteogenesis than group A and worse than group C. This might be since group B was injected with BMSC sheets in the distraction gap, which preserved the intercellular junctions and extracellular matrix while increasing the local concentration and retention of BMSCs, which was consistent with the experimental study by Ma et al. (7). Group C had good bone formation at all time points of the fixation. Gross specimen observation, Micro-CT, and histomorphological observation showed that the quality of new bone in the distraction gap was better in group C than in groups A and B at 3 and 6 weeks of fixation. These results indicated that local gene therapy of BMP-1-modified BMSC sheets might play an important role in promoting new bone formation in DO and compensate for the poor formation of bone scabs caused by rapid distraction. The limitation of the present study is that a positive control group (the deviation rate of 1 mm / D) was not provided. However, the related osteogenic indexes obtained in this study are basically consistent with the previous research results (7, 11, 18).

In summary, in the present study, with the combination of tissue and genetic engineering, the BMP-1-modified BMSC sheets were constructed. It was demonstrated that local injection of BMP-1-modified BMSC sheets could promote the formation and remodeling of new bone during rapid DO in the mandible, effectively compensating for the adverse effects of rapid distraction on new bone formation. The present method might improve the quality and efficiency of DO and shorten the treatment period, which might be an important guideline for further clinical applications.

## Data Availability Statement

The original contributions presented in the study are included in the article/supplementary material, further inquiries can be directed to the corresponding authors.

## Ethics Statement

The animal study was reviewed and approved by Ethics Committee of the Fourth Military Medical University.

## Author Contributions

Z-PS, L-SH, and J-LZ: conception and design of the research. YY and J-BL: acquisition of data. Y-JK: analysis and interpretation of the data. H-TS: statistical analysis. J-LZ: obtaining financing. Z-PS: writing of the manuscript. LT: critical revision of the manuscript for intellectual content. All authors have read and approved the final draft.

## Conflict of Interest

The authors declare that the research was conducted in the absence of any commercial or financial relationships that could be construed as a potential conflict of interest.

## Publisher's Note

All claims expressed in this article are solely those of the authors and do not necessarily represent those of their affiliated organizations, or those of the publisher, the editors and the reviewers. Any product that may be evaluated in this article, or claim that may be made by its manufacturer, is not guaranteed or endorsed by the publisher.
